# Evidence for a gene influencing heart rate on chromosome 5p13-14 in a meta-analysis of genome-wide scans from the NHLBI Family Blood Pressure Program

**DOI:** 10.1186/1471-2350-7-17

**Published:** 2006-03-01

**Authors:** Jason M Laramie, Jemma B Wilk, Steven C Hunt, R Curtis Ellison, Aravinda Chakravarti, Eric Boerwinkle, Richard H Myers

**Affiliations:** 1 Department of Neurology, Boston University School of Medicine, Boston, MA, USA; 2 Department of Bioinformatics, Boston University, MA, USA; 3 Section of Preventive Medicine and Epidemiology, Department of Medicine, Boston University School of Medicine, Boston, MA, USA; 4 Cardiovascular Genetics, University of Utah, Salt Lake City, UT, USA; 5 Institute of Genetic Medicine, Johns Hopkins University School of Medicine, Baltimore, MD, USA; 6 Human Genetics Center, University of Texas-Houston Health Science Center, Houston, TX, USA

## Abstract

**Background:**

Elevated resting heart rate has been shown in multiple studies to be a strong predictor of cardiovascular disease. Previous family studies have shown a significant heritable component to heart rate with several groups conducting genomic linkage scans to identify quantitative trait loci.

**Methods:**

We performed a genome-wide linkage scan to identify quantitative trait loci influencing resting heart rate among 3,282 Caucasians and 3,989 African-Americans in three independent networks comprising the Family Blood Pressure Program (FBPP) using 368 microsatellite markers. Mean heart rate measurements were used in a regression model including covariates for age, body mass index, pack-years, currently drinking alcohol (yes/no), hypertension status and medication usage to create a standardized residual for each gender/ethnic group within each study network. This residual was used in a nonparametric variance component model to generate a LOD score and a corresponding P value for each ethnic group within each study network. P values from each ethnic group and study network were merged using an adjusted Fisher's combining P values method and the resulting P values were converted to LOD scores. The entire analysis was redone after individuals currently taking beta-blocker medication were removed.

**Results:**

We identified significant evidence of linkage (LOD = 4.62) to chromosome 10 near 142.78 cM in the Caucasian group of HyperGEN. Between race and network groups we identified a LOD score of 1.86 on chromosome 5 (between 39.99 and 45.34 cM) in African-Americans in the GENOA network and the same region produced a LOD score of 1.12 among Caucasians within a different network (HyperGEN). Combining all network and race groups we identified a LOD score of 1.92 (*P *= 0.0013) on chromosome 5p13-14. We assessed heterogeneity for this locus between networks and ethnic groups and found significant evidence for low heterogeneity (*P *≤ 0.05).

**Conclusion:**

We found replication (LOD > 1) between ethnic groups and between study networks with low heterogeneity on chromosome 5p13-14 suggesting that a gene in this region influences resting heart rate.

## Background

Heart rate has been implicated as a risk factor in cardiovascular disease (CVD) [1-9], cancer [10,11], and sudden death in middle-aged men [12]. Furthermore, elevated heart rate has been shown to be an independent predictor of CVD[8,10,13]. Even after other risk factors have been controlled for, resting heart rate remains significantly associated with cardiovascular mortality and total mortality [10].

Singh et al. [14] and more recently Kupper et al. [15] reported that there is a significant genetic component to heart rate variability. Moreover, loci that influence heart rate have been located in a number of organisms including humans [16,17], mice [18], rats [19-21] and drosophila [22]. In a population of normotensive and hypertensive individuals Martin et al [23] report significant evidence of linkage (LOD = 3.9) on chromosome 4q for resting heart rate. On chromosome 10, a serine/glycine (Ser49Gly) substitution polymorphism in the β1 adrenergic receptor was found to be highly associated with heart rate in a Chinese and Japanese hypertensive cohort, with higher mean heart rates occurring in individuals that are serine homozygotes [24].

Due to its importance in predicting cardiovascular and all-cause mortality, we have used data from Caucasians and African-Americans enrolled in the NHLBI Family Blood Pressure Program to evaluate genetic factors associated with resting heart rate. We report a meta-analysis of genome scans within the GenNet, GENOA, and HyperGEN Networks, using genotyping carried out by the Mammalian Genotyping Service.

## Methods

### Study participants

Subjects were recruited by four multicenter networks investigating the genetics of hypertension/blood pressure (BP) known collectively as the Family Blood Pressure Program (FBPP)[25]. Data from 3 of the 4 networks [GenNet[26], GENOA [27], and HyperGEN [28] were appropriate for the present analysis. GenNet sampled Caucasian and African American families through mostly unmedicated probands with elevated BP levels who were younger than the age typically associated with the onset of hypertension. GENOA sampled 553 Caucasian and African American families with at least two full siblings with essential hypertension diagnosed before the age of sixty. HyperGEN sampled Caucasian and African American families that contained hypertensive sibships.

The present analyses were based on a total of 7,271 subjects. There were 542 Caucasians (from 203 families) and 776 African-Americans (from 281 families) in GenNet; 1,400 Caucasians (from 481 families) and 1,695 African Americans (from 547 families) in GENOA; and 1,340 Caucasians (from 583 families) and 1,518 African Americans (from 684 families) in HyperGEN who had both genotyping and data on resting heart rate and other covariates for adjustment of the phenotype. Each network has been approved by the appropriate local Institutional Review Board and all participants provided informed written consent.

### Heart rate measurement

Prior to examination, participants were told to avoid caffeinated products such as tea, coffee, and chocolate as well as any other food and heavy physical activity for twelve hours. Clinical examinations all took place in the morning. After being seated for five minutes, heart rate and blood pressure were measured by arterial pulsations measured from the upper arm using either a Dinamap or Omron automatic blood pressure monitor. A measure of heart rate derived from 24 hour monitoring would be ideal; however, the feasibility of such a measure on this number of participants is limited. GENOA and HyperGEN networks exclusively used the Dinamap monitor, whereas the GenNet network used both blood pressure monitors, with 71.3% of patients measured with a Dinamap monitor and 28.7% of patients measured with an Omron monitor. Each device was turned off and allowed to recalibrate between participants. Mean heart rate was calculated either from three (for the Dinamap monitor) or two (for the Omron monitor) measurements taken in a six minute interval to control for intra-individual variability.

### Genotyping

Blood was drawn into three 10 ml vacutainer tubes containing EDTA. Genomic DNA was isolated using standard protocols. The Mammalian Genotyping Service (MGS) in Marshfield, WI completed each genome screening using the same set of 368 highly polymorphic microsatellite markers for each network. These markers have an average heterozygosity of ~80%, an average intermarker distance of 10 cM, and cover ~95% of the human genome. Specific details on gel preparation, PCR conditions, and the genetic map were reported by Weber and Broman [29] and are available from the MGS website [30].

### Covariate measurement

Questionnaires were used to collect information on potentially confounding factors for heart rate including age, sex, smoking, alcohol use, and medication usage. Medication use was also assessed by inventory of the participant's current prescriptions. Height and weight for the calculation of body mass index (BMI) were ascertained during the participant's clinical examination. Smoking habits were defined by three categories: non-smoker, current smoker and past smoker and, if the participant answered yes to smoking currently or in the past, total pack years were calculated. Alcohol use was assessed by inquiring whether the person "presently drinks alcoholic beverages". Hypertension was designated for participants if they met any of three criteria: (1) currently taking anti-hypertension medication (2) average diastolic blood pressure ≥ 90 mmHg or (3) average systolic blood pressure ≥ 140 mmHg. In addition, normotensive individuals were defined as not being hypertensive or hypotensive.

Previous reports of the effect of different hypertension medication use on heart rate within the networks of the FBPP have shown that β-blocker medication users had, on average, a significantly lower heart rate than non-users[16]. Therefore, we adjusted heart rate for β-blocker medication use and excluded participants taking β-blocker medication in a separate analysis.

### Statistical methods

Mean values of resting heart rate were calculated from all available measurements; for the Dinamap monitor three readings were used and for the Omron monitor two measurements were used. We used a regression model to evaluate the relationship of covariates to heart rate within each gender and racial group. Variables that were significant predictors of heart rate were included in the race and gender-specific regression model for adjustment. They included age, age^2^, BMI, pack-years, currently drinking alcohol (yes/no), hypertension status and β-blocker use. We then calculated the standardized residuals of heart rate for each gender and racial group within each network (i.e. GenNet, GENOA and HyperGEN). A second standardized residual was generated after excluding β-blocker medication users from the sample and removing the β-blocker medication use covariate from the regression model. Residuals from the models described above were used in subsequent linkage analysis.

Linkage analyses were performed using variance component (VC) models as implemented in MERLIN version 0.10.2 [31]. Multipoint analysis was performed modeling genetic variance as additive polygenic and quantitative trait locus (QTL). The linkage analysis was carried out separately for each race within each network for all participants and for individuals not currently taking β-blocker medication.

P values obtained from MERLIN's VC linkage were combined using Fisher's method of combining P values [32]. Briefly, if *n *independent tests are made about the same hypothesis, resulting in the P values P_1_, P_2_... P_n _then ∑i=1n(−2ln⁡Pi)
 MathType@MTEF@5@5@+=feaafiart1ev1aaatCvAUfKttLearuWrP9MDH5MBPbIqV92AaeXatLxBI9gBaebbnrfifHhDYfgasaacH8akY=wiFfYdH8Gipec8Eeeu0xXdbba9frFj0=OqFfea0dXdd9vqai=hGuQ8kuc9pgc9s8qqaq=dirpe0xb9q8qiLsFr0=vr0=vr0dc8meaabaqaciaacaGaaeqabaqabeGadaaakeaadaaeWbqaaiabcIcaOiabgkHiTiabikdaYiGbcYgaSjabc6gaUjabdcfaqnaaBaaaleaacqWGPbqAaeqaaOGaeiykaKcaleaacqWGPbqAcqGH9aqpcqaIXaqmaeaacqWGUbGBa0GaeyyeIuoaaaa@3CB5@ is distributed as a χ^2 ^with 2*n *degrees of freedom. Combined P Values where converted to LOD scores using the equation P = 1 - Φ [sign (*LOD*) (2ln⁡(10)|LOD|)
 MathType@MTEF@5@5@+=feaafiart1ev1aaatCvAUfKttLearuWrP9MDH5MBPbIqV92AaeXatLxBI9gBaebbnrfifHhDYfgasaacH8akY=wiFfYdH8Gipec8Eeeu0xXdbba9frFj0=OqFfea0dXdd9vqai=hGuQ8kuc9pgc9s8qqaq=dirpe0xb9q8qiLsFr0=vr0=vr0dc8meaabaqaciaacaGaaeqabaqabeGadaaakeaadaGcaaqaaiabcIcaOiabikdaYiGbcYgaSjabc6gaUjabcIcaOiabigdaXiabicdaWiabcMcaPiabcYha8jabdYeamjabd+eapjabdseaejabcYha8jabcMcaPaWcbeaaaaa@3C1A@], where Φ is the cumulative Gaussian distribution function [33]. In addition, any LOD score derived from the above equation that was less than zero was converted to zero. Fisher's method was adjusted for non-parametric linkage where a LOD score of zero was interpreted as a P value of 12ln⁡(2)
 MathType@MTEF@5@5@+=feaafiart1ev1aaatCvAUfKttLearuWrP9MDH5MBPbIqV92AaeXatLxBI9gBaebbnrfifHhDYfgasaacH8akY=wiFfYdH8Gipec8Eeeu0xXdbba9frFj0=OqFfea0dXdd9vqai=hGuQ8kuc9pgc9s8qqaq=dirpe0xb9q8qiLsFr0=vr0=vr0dc8meaabaqaciaacaGaaeqabaqabeGadaaakeaadaWcaaqaaiabigdaXaqaaiabikdaYiGbcYgaSjabc6gaUjabcIcaOiabikdaYiabcMcaPaaaaaa@3408@ ≈ .72 [34].

A test for heterogeneity was performed using the program Heterogeneity and Genome Search Meta-analysis (HEGESMA)[35,36]. The genome was equally divided into 95 bins of ~30 cM and the maximum LOD score was recorded for each bin. Each bin was ranked separately in each network and ethnic group with the highest LOD score having the highest rank and heterogeneity was estimated by the Q statistic[35]. Statistical significance for observing low heterogeneity (*P *≤ 0.05) was estimated from a null distribution of the Q statistic calculated from 10,000 rank permutations within each study network and ethnic group.

## Results

Table 1 shows descriptive statistics for heart rate and the covariates included in the regression model to define the heart rate phenotype for all participants by network, race and gender. Unadjusted mean heart rates are higher among women than men in both ethnic groups in all networks. Furthermore, African-Americans had higher resting heart rates than Caucasians at 71.6 ± 2.7 and 67.9 ± 2.7 respectively. The GenNet network had higher heart rate readings than comparable ethnic/gender groups in both GENOA and HyperGEN. A gender effect for BMI was much greater in African-Americans than in Caucasians. Caucasian males and females had the same average BMI of 29.4 whereas African American males, on average, had lower BMI then females at 28.4 and 32.6, respectively. The prevalence of β-blocker medication usage was higher in Caucasians than in African-Americans. GENOA and HyperGEN had more individuals diagnosed with hypertension than GenNet.

**Table 1:** Demographic Information

Characteristics	GenNet			
	
	CA		AA	
		
	Male (n = 255)	Female (n = 287)	Male (n = 319)	Female (n = 457)
Age (years)	36.7(9.0)	37.5(9.0)	38.7(10.1)	41.6(12.1)
Body Mass index (kg/m2)	28.6(5.4)	28.5(6.7)	27.1(8.6)	32.1(8.6)
Resting Heart Rate (beats/minute)	68.2(9.4)	71.9(9.3)	74.3(12.4)	74.6(11.0)
Pack years of smoking	9.1(15.9)	6.1(10.6)	9.9(12.9)	6.6(10.7)
Current Alcohol Drinking (%)	71.4	61	43.9	22.3
β-Blocker users (%)	2.3	4.9	1.8	3.3
Hypertension status (%)				
Non-hypertensive	76.5	82.2	69	62.8
Hypertensive	23.5	17.8	31	37.2
Secondary	0	0	0	0

	GENOA			
	
	CA		AA	
		
	Male (n = 638)	Female (n = 762)	Male (n = 527)	Female (n = 1168)

Age (years)	55.6(10.8)	55.2(11)	58.4(9.9)	56.9(10.4)
Body Mass index (kg/m2)	30.3(5.1)	30.2(7)	28.4(4.9)	32.1(6.9)
Resting Heart Rate (beats/minute)	64.5(11)	67.8(11)	68.1(11.5)	69.6(10.9)
Pack years of smoking	20.2(9)	9(16.8)	17.4(20.5)	6.1(12.9)
Current Alcohol Drinking (%)	75.9	37.8	46.5	31.1
β-Blocker users (%)	24	23.3	9.5	10.1
Hypertension status (%)				
Non-hypertensive	24.8	27.8	31.1	26
Hypertensive	70.2	66.9	66.6	73.5
Secondary	5	5.3	2.3	0.4

	HyperGEN			
	
	CA		AA	
		
	Male (n = 638)	Female (n = 702)	Male (n = 518)	Female (n = 1000)

Age (years)	56.3(13.1)	56.6(12.3)	47.9(12.7)	48.0(12.9)
Body Mass index (kg/m2)	29.5(4.7)	29.5(6.6)	29.8(6.2)	33.6(8.1)
Resting Heart Rate (beats/minute)	65.4(10.8)	69.6(11.2)	69.9(13.5)	72.8(12.0)
Pack years of smoking	16.4(23.9)	8.2(17.2)	13.2(17.3)	6.6(13.3)
Current Alcohol Drinking (%)	40.3	24.5	42.3	16.5
β-Blocker users (%)	21.9	22.1	7.1	12.2
Hypertension status (%)				
Non-hypertensive	25.6	26.2	24.5	18.4
Hypertensive	74.5	73.1	75.5	81.6
Secondary	0	0.4	0	0

Demographic information by network, race (CA for Caucasian and AA for African-American) and medication use. Means (± standard deviation) are given.

Heritability estimates for adjusted heart rate within each network and ethnic group are shown in Table 2. Mean heritability estimates across all networks for all participants were 32.62 (SE = 2.53) for Caucasians and 27.41 (SE = 2.11) for African-Americans. For just those participants not taking β-blocker medication, the mean heritability estimates were 32.96 (SE = 2.34) for Caucasians and 33.14 (SE = 2.17) for African-Americans.

**Table 2:** Heritability and Medication Usage Data

	GenNet		GENOA		HyperGEN	
	
	CA	AA	CA	AA	CA	AA
All Participants	28.23%	28.82%	37.00%	30.15%	32.62%	23.27%
Participants not taking β-Blocker Medication	28.70%	29.27%	36.76%	30.61%	33.40%	29.02%

Heritability estimates (h^2^) for resting heart rate, by network, race (CA for Caucasian and AA for African-American) and medication use.

In Caucasians, the maximal LOD score of 2.06 was seen on chromosome 10 at 142.78 cM within the HyperGEN Network. In African-Americans, the maximal LOD score was seen in the GENOA Network, where chromosome 13 produced a LOD of 3.07 across a region from 55.31 cM to 63.9 cM. Excluding participants taking β-blocker medication had a minimal impact in LOD scores (average increase of 0.002). In the HyperGEN network the multipoint LOD score of 2.06 detected for Caucasians on chromosome 10 at 142.78 cM increased to 4.62 (microsatellites at this linkage peak are ATA29C03, GATA48G07A and GGAA5D10) and the maximal LOD score seen in African-Americans in GENOA network on chromosome 13 decreased from 3.07 to 1.48 after removal of individuals using β-blocker medication. In African-Americans, the maximal LOD score in the sample not taking β-blocker medication was on chromosome 16 at 43.89 cM in HyperGEN with a LOD of 2.48, an increase from 1.86. Two networks (GENOA and HyperGEN) showed replication (LOD score > 1.0) across different ethnic groups on chromosome 5 between 39.99 and 45.34 cM at the microsatellites GATA145D09 and GATA7C06. Table 3 summarizes all multipoint LOD scores greater than 1.0 within any network/race group in study participants that are not taking β-blocker medication and includes the position and marker name where the maximal LOD score occurred for resting heart rate (for a complete listing of all participants see Additional file 1).

**Table 3:** Genome Scan Multipoint LOD scores

			GenNet		GENOA		HyperGEN		Meta-Analysis		
			
Chr	Dist.	Marker	CA	AA	CA	AA	CA	AA	CA	AA	All
1	102.02	GATA61A06	0.12	1.03	0.17	0	0	0.26	0.04	0.65	0.49

3	44.81	GATA73D01	0.22	0	1.03	0	0	0	0.61	0.00	0.09
	70.61	ATA10H11	0.01	0	1.39	0.03	0	0.04	0.61	0.00	0.28
	89.91	AFM306YG5	0.49	0	0.23	0.11	0	1.29	0.27	0.68	0.82
	90.01	GATA148E04	0.51	0	0.23	0.11	0	1.30	0.28	0.69	0.84
	91.18	AFM191YG5	0.40	0	0.17	0.04	0	1.29	0.18	0.60	0.64
	91.28	UT7805	0.39	0	0.16	0.03	0	1.28	0.17	0.57	0.61
	176.54	GATA3H01	0	0.33	0	0.38	1.87	0.17	0.79	0.58	1.23
	181.87	GATA92B06	0	0	0.04	0.01	1.29	0	0.60	0.00	0.15
	215.84	ATA22E01	0	0	1.12	0	0.20	0.87	0.66	0.18	0.69
	224.88	AFM254VE1	0	0	0.13	0	0.42	1.94	0.16	0.83	0.81

4	104.94	GATA2F11	0	0.45	0.17	0	0	1.25	0.00	0.97	0.50

5	36.25	GATA134B03	0.15	0	0.17	1.46	0.75	0	0.70	0.51	1.08
	**39.99**	**GATA145D09**	**0.29**	**0**	**0.14**	**1.86**	**1.12**	**0.04**	**1.09**	**0.99**	**1.92**
	**45.34**	**GATA7C06**	**0.25**	**0**	**0.14**	**1.07**	**1.08**	**0.46**	**1.02**	**0.85**	**1.70**
	59.3	GATA21D04	0	0	0.77	0.75	0.61	1.48	0.75	1.41	1.97
	116.98	GATA68A03	0.14	0.01	0.05	0.22	0.19	1.33	0.18	0.98	0.95

6	128.93	GATA23F08	0	0.99	0	1.12	0	0	0.00	1.32	0.46
	137.74	GATA32B03	0	1.27	0	0.43	0	0	0.00	0.97	0.26
	146.06	GATA184A08	0	1.97	0	0	0	0	0.00	0.86	0.20
	154.64	GATA165G02	0	1.31	0.21	0	0	0	0.00	0.42	0.18

7	41.69	AFM224XG5	0	0.07	0	1.26	0	0	0.00	0.62	0.10
	41.79	GGAA3F06	0	0.07	0	1.26	0	0	0.00	0.62	0.10
	57.79	GATA31A10	0.01	0.01	0	1.54	0.01	0	0.00	0.71	0.29
	69.56	GATA24D12	0.05	0	0.01	1.24	0.06	0	0.02	0.38	0.26
	90.95	GATA73D10	0	0	0.01	1.21	0.76	0	0.23	0.36	0.49
	98.44	AFM165YH12	0.01	0	0.11	1.25	0.66	0	0.40	0.39	0.68
	98.54	GATA3F01	0.01	0	0.11	1.24	0.66	0	0.40	0.38	0.68

8	26.43	GATA23D06	0	0.01	0	0.34	0	1.68	0.00	1.37	0.49
	94.08	GATA14E09	0.22	0	0	1.1	0	0	0.00	0.30	0.11
	135.08	GATA7G07	0	1.28	0.33	0	0	0	0.00	0.40	0.22
	164.47	UT721	0.13	0	0	0	1.11	0	0.58	0.00	0.08

10	4.32	GATA88F09	0	0	0	1.15	0	0.46	0.00	0.91	0.23
	28.31	ATA31G11	0	0	0	0.67	0	1.17	0.00	1.10	0.33
	125.41	GATA64A09	0.01	0	0	0	1.26	0	0.52	0.00	0.06
	134.7	GATA48G07A	0	0.18	0	0.01	2.78	0	1.45	0.00	0.96
	142.78	ATA29C03	0.75	0.09	0	0.05	4.62	0	4.08	0.00	3.06
	148.17	GGAA5D10	0.48	0	0	0.23	3.66	0	2.96	0.00	2.05
	157.89	AFM212XD6	0.24	0.06	0	0.53	1.65	0	1.08	0.17	1.01
	170.94	AFM198ZB4	0.08	0.05	0	1.25	0.01	0	0.00	0.58	0.28

11	76.13	GATA90D07	0.05	0.43	0.08	0.46	0	1.19	0.00	1.61	1.05

12	17.72	GATA49D12	0	0	0	0.65	1.15	0	0.33	0.09	0.31
	19.68	M273ZC9	0	0	0	0.68	1.06	0	0.28	0.10	0.29
	78.14	GATA73H09	1.36	0	0	0.37	0	0	0.45	0.01	0.27
	80.52	GATA3F02	1.05	0	0	0.52	0	0	0.27	0.04	0.22
	136.82	GATA4H01	0.01	0	0	0	1.67	0.42	0.80	0.02	0.53
	149.6	GATA32F05	0	0	0	0.08	0.13	1.42	0.00	0.74	0.32
	160.68	ATA29A06	0	0	0	0.48	0	1.48	0.00	1.18	0.38

13	45.55	GATA29A09	0	0.21	0	1.78	0	0.12	0.00	1.50	0.57
	55.31	GATA64F08	0.05	0.11	0	1.48	0	0.12	0.00	1.15	0.51
	63.9	GATA7G10	0	0.02	0	1.73	0	0	0.00	0.86	0.21

14	44.06	GATA4B04	0	0	0	0.45	0	1.32	0.00	1.03	0.29
	105	AFM304YA5	1.03	0	0	0	0	0	0.26	0.00	0.00
	105.53	GGAA21G11	1.06	0	0	0	0	0	0.28	0.00	0.00

16	29.97	GATA42E11	0	0	0	0	0	1.08	0.00	0.29	0.00
	32.07	AFMB337ZC9	0	0	0	0	0	1.36	0.00	0.45	0.04
	43.89	AFM049XD2	0.02	0	0	0	0.01	2.48	0.00	1.23	0.63
	50.6	GATA71H05	0.06	0	0	0	0.06	1.65	0.00	0.64	0.35
	124.73	GATA11C06	0.53	0	0.08	0.04	0	1.60	0.18	0.81	0.82
	130.41	GATA71F09	0.11	0	0	0.22	0	2.28	0.00	1.54	0.84

17	66.85	ATC6A06	0	1.16	0	0.01	0.06	0	0.00	0.46	0.12

19	36.22	AFM224YE9	0	0	0	1.05	0	0	0.00	0.27	0.00
	42.28	AFM256YC9	0.03	0	0.41	1.07	0	0.07	0.09	0.49	0.44
	42.38	GATA66B04	0.03	0	0.42	1.06	0	0.07	0.09	0.49	0.44

21	2.99	GATA11C12	0	0	1.07	0	0	0	0.28	0.00	0.00
	13.05	GGAA3C07	0.02	0	1.11	0	0.04	0	0.64	0.00	0.10
	19.39	AFM344WF5	0	0	1.60	0	0.01	0	0.75	0.00	0.15
	24.73	GATA129D11	0	0	1.50	0	0	0	0.54	0.00	0.07
	27.4	AFM211ZG9	0	0	1.83	0.01	0.01	0	0.91	0.00	0.32
	35.45	AFM261ZG1	0	0	1.88	0	0.02	0.01	0.97	0.00	0.35
	36.77	ATA27F01	0	0	1.75	0	0.02	0.03	0.88	0.00	0.32
	40.49	GATA188F04	0	0	2.29	0	0.02	0.04	1.28	0.00	0.58
	45.87	AFM234XG9	0	0.01	2.25	0	0.01	0.03	1.22	0.00	0.65

Multipoint LOD scores greater than 1.0 for individuals not taking β-blocker medication. Each network is shown by race (CA for Caucasian and AA for African-American). LOD scores replicated between 2 networks are shown in bold. Distance is given in cM.

**Figure 1:**
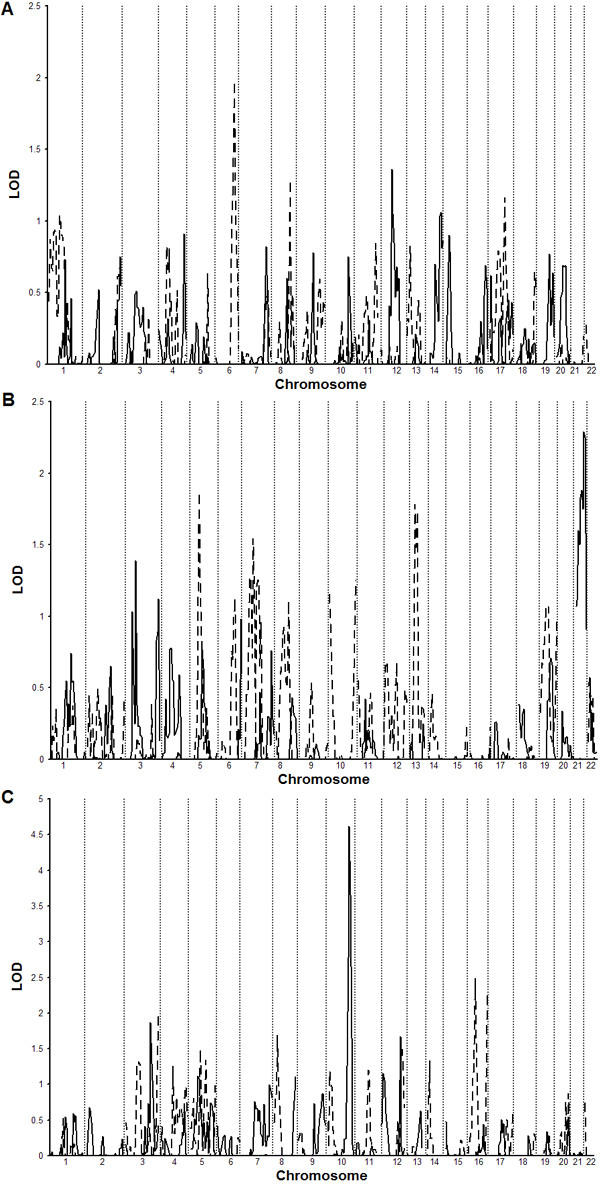
**Original Genome Scans for Each Network. **Genome scans separated by race for participants not taking β-blocker medication across all three networks. Caucasians and African-Americans are represented in each panel by a solid and a dashed line respectively. A) GenNet B) GENOA C) HyperGEN

**Figure 2:**
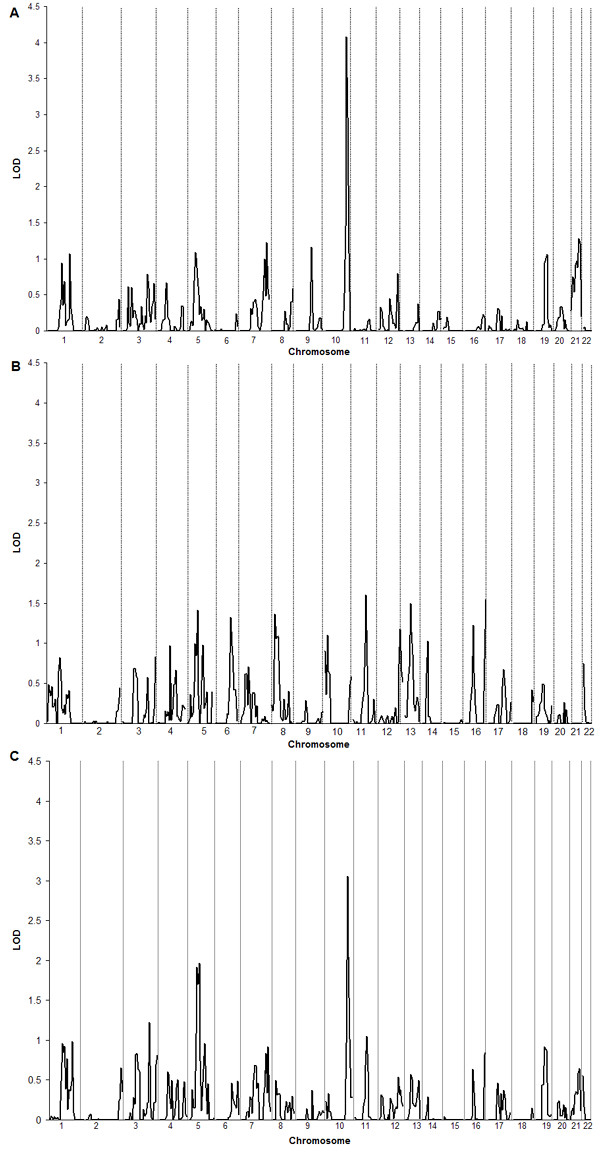
**Meta-analysis of LOD scores. **Meta-analysis of multipoint LOD scores for all genome scans for participants not taking β-blocker medication across all three networks. A) Caucasians B) African-Americans C) Combined

**Figure 3:**
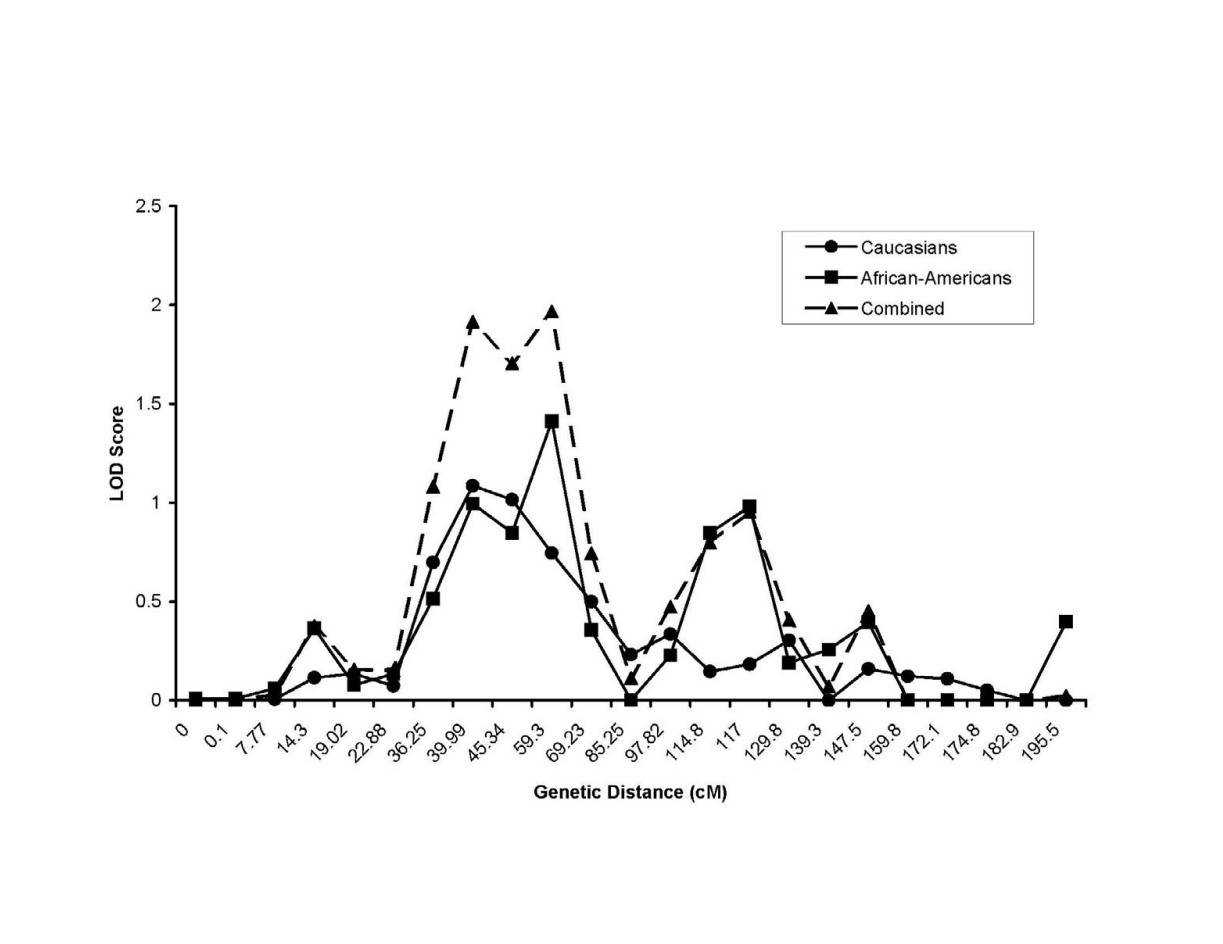
**Chromosome 5 meta-analysis of LOD scores. **Meta-analysis of multipoint LOD scores on chromosome 5 for resting heart rate for all Caucasians not taking β-blocker medication (Caucasians) and all African-Americans not taking β-blocker medication (African-Americans). Also shown is the combined race meta-analysis for all individuals not taking β-blocker medication (Combined)

Figure 1 shows the original genome scan for each network from participants not taking β-blocker medication separated by ethnic group. Figure 2 shows the meta-analysis results from participants not taking β-blocker medication combining the genome scans of all networks by race and combing all races and networks. The combined Caucasian sample not taking β-blocker medication showed seven LOD scores greater than 1.0 with a maximum of 4.08 on chromosome 10 at 142.8 cM near the microsatellite ATA29C03. The combined African-American sample not taking β-blocker medication showed nine LOD scores above 1.0 with a maximum of 1.60 on chromosome 11 at 76.13 cM (GATA90D07). When ethnic groups where combined there were two major peaks seen with a maximal LOD score of 3.06 on chromosome 10 at 78.14 cM and a smaller LOD score of 1.96 on chromosome 5 between 39.99 and 45.34 cM. Figures 2 and 3 show chromosomes 5 and 10, respectively, presenting the meta-analysis by ethic group and the total combined meta-analysis in individuals not taking β-blocker medication. We found significance evidence of low heterogeneity (*P *≤ 0.05) for chromosome 5 between 39.99 and 45.34 cM.

## Discussion

Resting heart rate is a clinically significant variable that a large number of studies have shown to be associated with CVD, atherosclerosis, cancer, and all-cause mortality[1,2,5,6,8,10,11,37-39]. Individuals with elevated resting heart rate have been shown to have high cholesterol, triglycerides and fasting insulin which are known risk factors for hypertension[40]. Furthermore, elevated resting heart rate has been associated with increased BMI and blood glucose, dyslipidemia, and high hematocrit[37,41].

The pathogenesis of the connection between tachycardia-associated disease and death is not well understood. However, it has been postulated that tachycardia is a marker of abnormal autonomic control and is caused by shift in sympathovagal balance towards relative sympathetic dominance [40]. Given the importance of heart rate as an independent predictor of CVD and death, it becomes increasingly important to identify genetic modifiers that affect heart rate. It has been shown that heart rate lowered by either surgical (ablation of the sinoatrial node [42]) or pharmaceutical (administration of β-blocker medications[43,44]) means in cholesterol-fed monkeys slowed the development of coronary atherosclerosis. Therefore the identification of genes influencing heart rate may produce new drug targets that could decrease atherosclerosis and ultimately CVD.

We estimated the heritability of resting heart rate across all networks for all participants to be 32.62 % (SE = 2.53) for Caucasians and 27.41 % (SE = 2.11) for African-Americans. These values are slightly higher than previous estimates of 26% [23] and 21% [14] but lower than a twin study estimate of 59% [45]. Additionally much higher correlation for heart rate among siblings (0.23) than spouse pairs (0.06) has been reported [14]. This evidence suggests a strong genetic component to heart rate and heart rate variability.

In our genome scan for resting heart rate, we found suggestive evidence for linkage at several loci for both Caucasian and African Americans, but only one locus in Caucasians not taking β-blockers reached the level of genomewide significance with a LOD score of 4.62 (chromosome 10, 142.78 cM). Combining all races and networks we found suggestive linkage at several loci including overlapping regions of linkage across races in two separate networks, GENOA and HyperGEN, implicating chromosome 5 with LOD scores of 1.86 and 1.12 respectively.

The largest meta-analysis peak was seen on chromosome 10 at 142.78 cM and seems to be driven entirely by Caucasians with a nominal contribution of the African-American cohort. The second largest meta-analysis peak was seen on chromosome 5 between 39.99 and 45.34 cM and is driven by two completely separate networks and ethnic groups. This linkage peak between 39.99 and 45.34 cM overlaps with a linkage peak in a recent genome scan for neonatal atrial fibrillation [46]. Contrary to previous reports [16,23], we saw no evidence for a QTL on chromosome 4q. Finally, while the HyperGEN study provided strong evidence for linkage on chromosome 10 in Caucasians and in the meta-analysis, the locus was only replicated with a 0.75 LOD in one other race/network group. While past studies of this region on chromosome 10, have reported association for a polymorphism in the β adrenergic receptor with increased resting heart rate[24], the evidence for linkage to this region was driven by a single race/network group in our study.

The major strengths of our analysis include that it was carried out in multiple networks, each with multiple sites across the country, using a large population of Caucasian and African Americans. Moreover, adjustments for factors known to affect heart rate (including BMI, smoking status, medication and alcohol use) were made prior to analysis.

## Conclusion

Our finding of linkage in several locations in the genome strongly suggests that heart rate is a polygenic phenotype and additional study of the implicated loci is needed. Notably, the overlapping linkage region (LOD > 1) across ethnic groups and study centers on chromosome 5p13-14 with low heterogeneity provides strong support for a QTL influencing elevated resting heart rate.

## Competing interests

The author(s) declare that they have no competing interests.

## Authors' contributions

JML assembled the genotype and phenotype data from the multiple networks and carried out the genome-scans and meta-analysis and drafted the manuscript. JBW conceived the study and assisted in assembling the genotype and phenotype data from the multiple networks and helped to draft the manuscript. SCH is the director of HyperGEN network and helped to finalize the manuscript. RCE is the director of the Framingham field center for HyperGEN data collection and helped to finalize the manuscript. AC is the main contact for the GenNet network and helped to finalize the manuscript. EB is director of the GENOA network and helped to finalize the manuscript. RHM assisted in data analysis and helped draft the manuscript. All authors have read and approved the final version of the manuscript.

**Figure 4:**
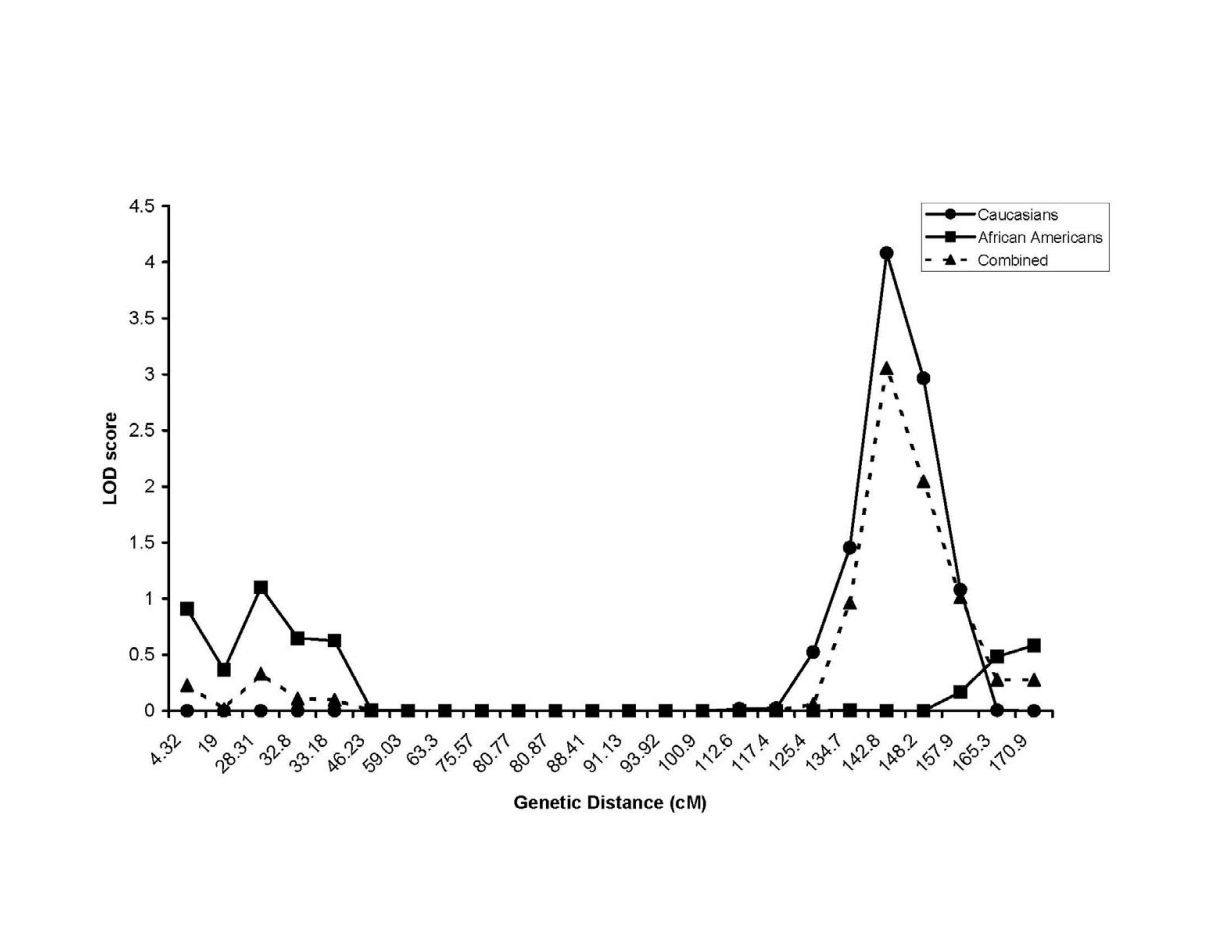
**Chromosome 10 meta-analysis of LOD scores. **Meta-analysis of multipoint LOD scores on Chromosome 10 for resting heart rate for all Caucasians not taking β-blocker medication (Caucasians) and all African-Americans not taking β-blocker medication (African-Americans). Also shown is the combined race meta-analysis for all individuals not taking β-blocker medication (Combined)

## Pre-publication history

The pre-publication history for this paper can be accessed here:



## Supplementary Material

Additional File 1 **Multipoint LOD scores for all study participants **Each network is shown by race (CA for Caucasian and AA for African-American). Distance is given in cM. This is an Excel™ file and Microsoft^® ^Excel™ should be used for viewing. Click here for file

## References

[B1] Dyer AR, Persky V, Stamler J, Paul O, Shekelle RB, Berkson DM, Lepper M, Schoenberger JA, Lindberg HA (1980). Heart rate as a prognostic factor for coronary heart disease and mortality: findings in three Chicago epidemiologic studies. Am J Epidemiol.

[B2] Kannel WB, Kannel C, Paffenbarger RSJ, Cupples LA (1987). Heart rate and cardiovascular mortality: the Framingham Study. Am Heart J.

[B3] Gillman MW, Kannel WB, Belanger A, D'Agostino RB (1993). Influence of heart rate on mortality among persons with hypertension: the Framingham Study. Am Heart J.

[B4] Habib G (1997). Reappraisal of the importance of heart rate as a risk factor for cardiovascular morbidity and mortality. Clin Ther.

[B5] Greenland P, Daviglus ML, Dyer AR, Liu K, Huang CF, Goldberger JJ, Stamler J (1999). Resting heart rate is a risk factor for cardiovascular and noncardiovascular mortality: the Chicago Heart Association Detection Project in Industry. Am J Epidemiol.

[B6] Palatini P, Julius S (1999). Relevance of heart rate as a risk factor in hypertension. Curr Hypertens Rep.

[B7] Palatini P (1999). Heart rate as a risk factor for atherosclerosis and cardiovascular mortality: the effect of antihypertensive drugs. Drugs.

[B8] Palatini P, Casiglia E, Julius S, Pessina AC (1999). High heart rate: a risk factor for cardiovascular death in elderly men. Arch Intern Med.

[B9] Palatini P (2001). Heart rate as a cardiovascular risk factor: do women differ from men?. Ann Med.

[B10] Kristal-Boneh E, Silber H, Harari G, Froom P (2000). The association of resting heart rate with cardiovascular, cancer and all-cause mortality. Eight year follow-up of 3527 male Israeli employees (the CORDIS Study). Eur Heart J.

[B11] Persky V, Dyer AR, Leonas J, Stamler J, Berkson DM, Lindberg HA, Paul O, Shekelle RB, Lepper MH, Schoenberger JA (1981). Heart rate: a risk factor for cancer?. Am J Epidemiol.

[B12] Jouven X, Zureik M, Desnos M, Guerot C, Ducimetiere P (2001). Resting heart rate as a predictive risk factor for sudden death in middle-aged men. Cardiovasc Res.

[B13] Hjalmarson A (1998). Significance of reduction in heart rate in cardiovascular disease. Clin Cardiol.

[B14] Singh JP, Larson MG, O'Donnell CJ, Tsuji H, Evans JC, Levy D (1999). Heritability of heart rate variability: the Framingham Heart Study. Circulation.

[B15] Kupper NH, Willemsen G, van den Berg M, de Boer D, Posthuma D, Boomsma DI, de Geus EJ (2004). Heritability of Ambulatory Heart Rate Variability. Circulation.

[B16] Wilk JB, Myers RH, Zhang Y, Lewis CE, Atwood L, Hopkins PN, Ellison RC (2002). Evidence for a gene influencing heart rate on chromosome 4 among hypertensives. Hum Genet.

[B17] Singh JP, Larson MG, O'Donnell CJ, Tsuji H, Corey D, Levy D (2002). Genome scan linkage results for heart rate variability (the Framingham Heart Study). Am J Cardiol.

[B18] Sugiyama F, Churchill GA, Li R, Libby LJ, Carver T, Yagami K, John SW, Paigen B (2002). QTL associated with blood pressure, heart rate, and heart weight in CBA/CaJ and BALB/cJ mice. Physiol Genomics.

[B19] Alemayehu A, Breen L, Krenova D, Printz MP (2002). Reciprocal rat chromosome 2 congenic strains reveal contrasting blood pressure and heart rate QTL. Physiol Genomics.

[B20] Jaworski RL, Jirout M, Closson S, Breen L, Flodman PL, Spence MA, Kren V, Krenova D, Pravenec M, Printz MP (2002). Heart rate and blood pressure quantitative trait loci for the airpuff startle reaction. Hypertension.

[B21] Kreutz R, Struk B, Stock P, Hubner N, Ganten D, Lindpaintner K (1997). Evidence for primary genetic determination of heart rate regulation: chromosomal mapping of a genetic locus in the rat. Circulation.

[B22] Ashton K, Wagoner AP, Carrillo R, Gibson G (2001). Quantitative trait loci for the monoamine-related traits heart rate and headless behavior in Drosophila melanogaster. Genetics.

[B23] Martin LJ, Comuzzie AG, Sonnenberg GE, Myklebust J, James R, Marks J, Blangero J, Kissebah AH (2004). Major quantitative trait locus for resting heart rate maps to a region on chromosome 4. Hypertension.

[B24] Ranade K, Jorgenson E, Sheu WH, Pei D, Hsiung CA, Chiang FT, Chen YD, Pratt R, Olshen RA, Curb D, Cox DR, Botstein D, Risch N (2002). A polymorphism in the beta1 adrenergic receptor is associated with resting heart rate. Am J Hum Genet.

[B25] (2002). Multi-center genetic study of hypertension: The Family Blood Pressure Program (FBPP). Hypertension.

[B26] Thiel BA, Chakravarti A, Cooper RS, Luke A, Lewis S, Lynn A, Tiwari H, Schork NJ, Weder AB (2003). A genome-wide linkage analysis investigating the determinants of blood pressure in whites and African Americans. Am J Hypertens.

[B27] Kardia SL, Rozek LS, Krushkal J, Ferrell RE, Turner ST, Hutchinson R, Brown A, Sing CF, Boerwinkle E (2003). Genome-wide linkage analyses for hypertension genes in two ethnically and geographically diverse populations. Am J Hypertens.

[B28] Rao DC, Province MA, Leppert MF, Oberman A, Heiss G, Ellison RC, Arnett DK, Eckfeldt JH, Schwander K, Mockrin SC, Hunt SC (2003). A genome-wide affected sibpair linkage analysis of hypertension: the HyperGEN network. Am J Hypertens.

[B29] Weber JL, Broman KW (2001). Genotyping for human whole-genome scans: past, present, and future. Adv Genet.

[B30] Mammalian Genotyping Services. http://research.marshfieldclinic.org/genetics.

[B31] Abecasis GR, Cherny SS, Cookson WO, Cardon LR (2002). Merlin--rapid analysis of dense genetic maps using sparse gene flow trees. Nat Genet.

[B32] Fisher RA (1950). Statistical methods for research workers.

[B33] Ott J (1985). Analysis of human genetic linkage.

[B34] Province MA (2001). The significance of not finding a gene. Am J Hum Genet.

[B35] Zintzaras E, Ioannidis JP (2005). Heterogeneity testing in meta-analysis of genome searches. Genet Epidemiol.

[B36] Zintzaras E, Ioannidis JP (2005). HEGESMA: genome search meta-analysis and heterogeneity testing. Bioinformatics.

[B38] Palatini P, Julius S (1997). Heart rate and the cardiovascular risk. J Hypertens.

[B39] Berton GS, Cordiano R, Palmieri R, Gheno G, Mormino P, Palatini P (2002). Heart rate during myocardial infarction: relationship with one-year global mortality in men and women. Can J Cardiol.

[B40] Fujiura Y, Adachi H, Tsuruta M, Jacobs DRJ, Hirai Y, Imaizumi T (2001). Heart rate and mortality in a Japanese general population: an 18-year follow-up study. J Clin Epidemiol.

[B41] Palatini P, Casiglia E, Pauletto P, Staessen J, Kaciroti N, Julius S (1997). Relationship of tachycardia with high blood pressure and metabolic abnormalities: a study with mixture analysis in three populations. Hypertension.

[B42] Palatini P, Julius S (1997). Association of tachycardia with morbidity and mortality: pathophysiological considerations. J Hum Hypertens.

[B43] Beere PA, Glagov S, Zarins CK (1984). Retarding effect of lowered heart rate on coronary atherosclerosis. Science.

[B44] Kaplan JR, Manuck SB, Adams MR, Weingand KW, Clarkson TB (1987). Inhibition of coronary atherosclerosis by propranolol in behaviorally predisposed monkeys fed an atherogenic diet. Circulation.

[B45] Kaplan JR, Manuck SB, Clarkson TB (1987). The influence of heart rate on coronary artery atherosclerosis. J Cardiovasc Pharmacol.

[B46] Jedrusik P, Januszewicz A, Busjahn A, Zawadzki B, Wocial B, Ignatowska-Switalska H, Berent H, Kuczynska K, Oniszczenko W, Strelau J, Luft FC, Januszewicz W (2003). Genetic influence on blood pressure and lipid parameters in a sample of Polish twins. Blood Press.

[B47] Oberti C, Wang L, Li L, Dong J, Rao S, Du W, Wang Q (2004). Genome-wide linkage scan identifies a novel genetic locus on chromosome 5p13 for neonatal atrial fibrillation associated with sudden death and variable cardiomyopathy. Circulation.

